# Effect of complete decongestive therapy and home program on health- related quality of life in post mastectomy lymphedema patients

**DOI:** 10.1186/s12905-016-0303-9

**Published:** 2016-05-04

**Authors:** Ganeswara Rao Melam, Syamala Buragadda, Adel A. Alhusaini, Nisha Arora

**Affiliations:** Department of Rehabilitation Sciences, College of Applied Medical Sciences, King Saud University, PO Box 10219, Riyadh, 11433 Saudi Arabia; Maharishi Markendeshwar Institute of Physiotherapy and Rehabilitation, M.M University, Mullana, India

**Keywords:** Health related quality of life, Functional status, Post mastectomy lymphedema

## Abstract

**Background:**

Secondary lymphedema is common in women treated for breast cancer. It may be a result of surgery or radiotherapy. Edema commonly affects the arm, leading to discomfort, reduced arm movements, pain and diminished quality of life. Therefore, the relationship between post mastectomy lymphedema and quality of life has evolved as an important criteria in treatment of breast cancer survivors.

**Methods:**

Sixty breast cancer survivors who developed post mastectomy lymphedema were recruited. Patients were divided into 2 groups (*n* = 30) according to the treatment they received; Conventional therapy (CT) and Complete Decongestive Therapy (CDT) groups. Measurements were taken at baseline, 4 and 6 weeks. Health related Quality of Life was evaluated with the EORTC QLQ C30 and EORTC QLQ-BR23 questionnaires. Pain was measured using the Visual Analogue Scale. Descriptive statistics were used to analyze participant demographics and repeated measures of ANOVA was used for within and between group comparisons.

**Results:**

Both groups showed improved quality of life and diminished pain after 6 weeks of treatment. However, greater improvement was observed in CDT group compared to the CT group.

**Conclusion:**

In this study, remedial exercises and home program in addition to manual lymphatic drainage and compression bandaging resulted in improved quality of life. Early identification of lymphedema and incorporation of remedial exercises and a home program improve the quality of life for breast cancer survivors.

**Trial registration:**

Trial registry ID: ISRCTN13242080, Date of registration: 7 April 2016

## Background

Health related quality of life (HRQOL) of patients is a topic of growing interest. HRQOL reflects the impact of disease and its treatment on functional health status (i.e., limitations in physical, psychological and social functioning) and global wellbeing [1]. Post mastectomy lymphedema (PML) is frequently encountered by breast cancer patients which in turn leads to poor functional recovery, chronic disability and impaired quality of life [2–4]. Women with arm edema secondary to breast cancer therapy may suffer from psychological morbidity such as anxiety and depression, functional and physical impairment, and diminished quality of life [5–7] Breast cancer symptoms, type of surgical intervention, adjuvant therapies and women’s economic level are directly associated with Health-Related Quality of Life [8–11]. Breast cancer survivors may find lymphedema more distressing than mastectomy, because hiding the physiological manifestations and loss of function is harder which in turn leads to decreased quality of life [12–16].

Several therapeutic interventions exist to treat this potentially distressing and disabling condition, but no consensus has been reached as to what constitutes optimal or definitive treatment of lymphedema [12, 13]. Complete decongestive therapy is considered the mainstay of lymphedema treatment. It consists of 4 components- 1) Manual lymphatic drainage (MLD), 2) compression bandaging, 3) exercises to enhance lymphatic drainage, and 4) skin care [17, 18]. MLD was developed in 1930s by Dr. Emil Vodder. It is a unique massage technique which uses specific hand movements to provide a gentle pumping action on the skin. The gentle, rhythmic, pumping, massage movements follow the direction of lymph flow and produce rapid results. This ensures that the maximum skin stretching effect is gained with the minimum of pressure. As a result, lymph flow improves without increasing capillary filtration [19].

MLD has been shown to have a number of physiological effects which include an increase in the contraction rate of lymph, increased reabsorption of proteins, reduced micro lymphatic hypertension and improved collateral lymph drainage between the lymphatic territories of the skin. Improved drainage enables fluid to be redirected away from edematous areas towards the functioning lymph nodes in unaffected areas, an important principle in lymphedema management [20, 21]. Patients’ education and self -care is important and considered critical for successful long-term effects. Clinicians require evidence to support the intensity of initial intensive treatment as well as the importance of each component of CDT. Improvements in the treatment of breast cancer have led to increased survival rates and increased emphasis on improving outcomes and quality of life through targeted rehabilitation [22, 23]. The evidence from cross-sectional and longitudinal studies on patients with lymphedema showed poor psychological adjustment, greater deficits in their ability to function physically and socially and increased anxiety and depression [7]. Several valid instruments exists to measure quality of life in breast cancer patients. The European Organization for Research and Treatment of Cancer Quality of Life Questionnaire (EORTC QLQ-C30) [24–27] and its breast cancer specific complementary measure (EORTC QLQ-BR23) [28–35], and the Functional Assessment Cancer Therapy General questionnaire (FACT-G) and its breast cancer module (FACT-B), are found to be the most common and well developed instruments to measure quality of life in breast cancer patients [36–38]. EORTC QLQ C30 consists of 30 functional, symptom and individual items designed to address a wide range of QOL issues relevant to a broad spectrum of cancer [24, 39]. EORTC QLQ-BR23 is also a reliable and valid supplementary measure of the quality of life in breast cancer patients and can be used in clinical trials and oncology studies [40]. Studies of quality of life can further indicate the directions needed for more efficient treatment of post mastectomy lymphedema. The aim of this study is to evaluate the effect of adding an exercise component and a home program to Complete Decongestive Therapy (CDT) on Health related quality of life in post-mastectomy lymphedema patients.”

## Methods

This is a mixed factorial design that includes both between and within subjects variables. Eighty subjects were screened and 20 individuals were excluded as they did not meet the inclusion criteria. A power analysis indicated that 27 participants per group would provide 80 % power to detect a treatment effect of 0.5. Sixty participants were divided into two groups: conventional treatment (CT) group and complete decongestive therapy (CDT) group. Each group comprised of 30 participants with a mean age of 56.3 ± 3.3 years and 56 ± 3.5 years respectively. Recruitment was done from Maharishi Markendeshwar University hospital. Participants’ were aged between 50 and 70 years, who had unilateral mastectomy for stage I and II breast cancer. They completed the radiotherapy and chemotherapy sessions. Arm circumferences were measured using a cloth measuring tape at four levels: the metacarpophalangeal joints, wrist joint, 15 cm distal to the lateral epicondyle, and 10 cm proximal to the lateral epicondyle. Arm volume was calculated using the formula” V = h (C12 + C1C2-C22)/12π^2^”. Subjects who developed lymphedema more than 3 cm compared to contralateral extremity were included. Participants with primary lymphedema, bilateral lymphedema, pulmonary edema, congestive heart failure or any contraindications limiting therapy were excluded. The purpose and procedure of the study was explained to all the participants. A signed informed consent was taken. Maharishi Markendeshwar University ethical committee approved the study. Systematic random sampling procedure was used to include the participants into the study. Data was collected every Monday and Wednesday from the physical therapy outpatient clinic. “Volunteer participants assessed on Monday were assigned to the CT group, whereas participants assessed on Wednesday were assigned to the CDT group. Demographic characteristics such as education level, marital status, type of surgery, and affected arm are presented in Table 1. The majority of the participants were literate and married. Lymphedema was observed in both dominant and non-dominant upper limbs. There were no significant differences in the demographic characteristics between the groups (*p* <0.05).

**Table 1:** Participant demographics

Variables		CT group (*n* = 30) M ± SD	CDT group (*n* = 30)	*t* Value	Sig.(*p* value)
Age (Years)		56.3 ± 3.3	56 ± 3.5	0.34	0.7*
Educational level	Literate	25 (42 %)	27 (45 %)	0.14	0.7*
	Illiterate	5 (8 %)	3 (5 %)		
Marital status	Single	4 (7 %)	5 (8 %)	0.13	0.7*
	Married	26 (43 %)	25 (42 %)		
Type of surgery	Radical Mastectomy	17 (43 %)	18 (45 %)	0.22	0.6*
	Modified Radical Mastectomy	3 (8 %)	2 (5 %)		
Affected Arm	Dominant	15 (38 %)	14 (35 %)	0.12	0.7*
	Non Dominant	5 (13 %)	6 (15 %)		

*M* mean, *SD* standard deviation, *t- value* independent *t* test, *Not significant at *P* ≤0.05

### Procedure

Conventional therapy (CT) group participants received manual lymphatic drainage, low elastic compression garment, gleno-humeral mobilization and deep breathing exercises. Massage strokes were applied to the side of the edematous limb, starting at the base of the neck and progressing to the affected limb. Massage was always directed proximally from the upper arm to the axilla, and then from the hand to the elbow. Finally, the whole limb was massaged from the distal to the proximal portion. CDT group received manual lymphatic drainage, compression garment worn 23 h daily, remedial exercises and a home program. Both groups received treatment 5 times a week for 6 weeks. CDT group participants and occasionally family members received training in self –massage. They were encouraged to do self -lymph drainage at least once daily. Remedial exercises were given with diaphragmatic breathing exercises in between. The following order was adopted for remedial exercises by a trained physiotherapist: 1) warm up activity by active mobilization of large joints at moderate pace for 5 min; 2) shoulder girdle mobilization-scapular retraction, protraction, depression, shoulder extension, elbow flexion and extension, wrist flexion and extension and ball squeeze; 3) Pectorals and trapezius muscles stretching. The patients were comfortably seated, relaxed, placed their hands over their abdominal muscles, and took deep breaths through the nose and a prolonged expiration through mouth without any strenuous effort (diaphragmatic breathing). The 1 h home program involved self -lymphatic drainage, skin care and the remedial exercises. Participants received booklets on the home program after initial education and training about home exercises. They were requested to keep a log for their home program. Both groups were treated for 6 weeks. Pain and QOL were assessed at baseline, 4th and 6th week of treatment.

The EORTC (European Organization of Research and Treatment for Cancer) QLQ-C30 version 3.0 is a 30-item core cancer specific questionnaire measuring QOL in cancer patients. This self-administered questionnaire incorporates five functional scales: Physical (PF), role (RF), cognitive (CF), emotional (EF) and social (SF), three symptom scales for fatigue, pain and nausea/vomiting, a global health QOL scale, and several single items for the perceived financial impact of disease and treatment and for the assessment of additional symptoms such as dyspnea, appetite loss, sleep disturbance, constipation and diarrhea which are commonly reported by cancer patients. All items were scored on 4-point Likert scales ranging from 1 (not at all) to 4 (very much). As an exception, item 29 and 30 in the global health QOL subscale were scored on a modified 7 point linear analogue scale. All functional scales and individual item scores were transformed to a 0–100 scale with higher values indicating a higher functioning in functional scales and an increased presence of symptoms in symptom scales. Approval was obtained from EORTC Quality of Life Group. The EORTC QLQ-BR23 is a 23-item breast cancer-specific questionnaire about the common side effects of therapy, body image, sexuality, and outlook for the future. All items were scored on 4-point Likert scales ranging from 1 (not at all) to 4 (very much). The scoring approach for the QLQ-BR23 is identical in principle to that for the function and symptom scales/single items of the QLQ-C30.

## Results

The data was analyzed using SPSS 16 software package with 95 % confidence interval. Descriptive statistics and *t*-test was used for subject’s demographic characteristics (Table 1). The level of significance was set at 0.05. Scores on each process measure were analyzed with a 2 × 3 mixed model ANOVA, with treatment group (conventional vs. complete decongestive therapy) serving as the between-subjects factor and time of assessment (baseline, 4th week and 6th week) serving as the repeated within-subjects factor. Table 2 shows values of outcome measures by treatment group and time of measurement.

**Table 2:** Values of outcome measures by treatment group and time of measurement

		CDT	CT
Outcome measure	Time of measurement	Mean ± SD (95 % CI)	Mean ± SD (95 % CI)
VAS	Baseline	6.87 ± 0.94 (6.52–7.22)	6.90 ± 1.09 (6.49–7.31)
	4th Week	3.17 ± 0.87 (2.84–3.49)	4.53 ± 1.07 (4.13–4.93)
	6th Week	1.40 ± 0.50 (1.21–1.59)	2.93 ± 0.87 (2.61–3.26)
EORTC QLQ C30 Global scale	Baseline	39.60 ± 4.74 (37.83–41.37)	39.63 ± 4.71 (37.81–41.39)
	4th Week	41.9 ± 4.72 (40.14–43.66)	40.57 ± 4.72 (38.80–42.33)
	6th Week	49.13 ± 5.49 (47.08–51.18)	41.57 ± 4.72 (39.80–43.33)
EORTC QLQ C30 Functional scale	Baseline	79.93 ± 1.41 (79.41–80.46)	79.93 ± 1.41 (79.41–80.46)
	4th Week	83.27 ± 1.98 (82.53–84.01)	80.87 ± 1.36 (80.36–81.37)
	6th Week	85.27 ± 1.98 (84.53–86.01)	81.83 ± 1.42 (81.30–82.36)
EORTC QLQ C30 Symptoms scale	Baseline	40.93 ± 3.26 (39.72–42.15)	40.93 ± 3.26 (39.72–42.15)
	4th Week	44.07 ± 3.42 (42.79–45.34)	41.93 ± 3.26 (40.72–43.15)
	6th Week	47.07 ± 3.51 (45.75–48.38)	42.90 ± 3.32 (41.66–44.14)
EORTC QLQ BR23 Functional scale	Baseline	32.20 ± 2.11 (31.41–32.99)	32.20 ± 2.11 (31.41–32.99)
	4th Week	36.37 ± 2.54 (35.42–37.31)	33.20 ± 2.11 (32.41–33.99)
	6th Week	38.13 ± 2.71 (37.12–39.15)	34.17 ± 2.20 (33.35–34.99)
EORTC QLQ BR23 Symptoms scale	Baseline	58.67 ± 2.34 (57.79–59.54)	58.67 ± 2.34 (57.79–59.54)
	4th Week	62.33 ± 2.52 (61.39–63.28)	59.67 ± 2.34 (58.79–60.54)
	6th Week	62.63 ± 7.99 (59.55–65.52)	60.63 ± 2.40 (59.74–61.53)

*CT* conventional therapy, *CDT* complete decongestive therapy, *VAS* visual analogue scale, *SD* standard deviation, *CI* confidence interval

### Pain

**Fig. 1:**
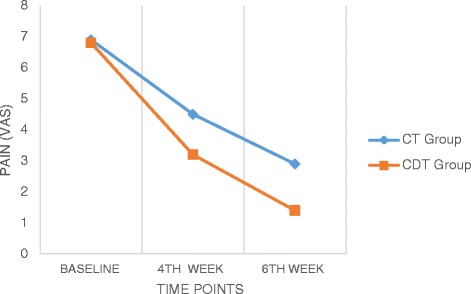
Comparison of Pain values within and between the groups

There was a significant difference across three time points, *F* = 991.96, *p* < 0.01 (Table 3) and significant differences between groups, *F* = 23.82, *p* < 0.01, in VAS (Table 4). There was also a significant interaction between time and group, *F* = 29.34, *p* < 0.01. This indicates that there were significant changes over time in VAS score across all samples and further analysis of interaction between time and group shows that changes in VAS over time are not equivalent across both the intervention groups (Fig. 1). Further between test subjects analysis indicates that group difference in VAS scores averaged across the time was significant. The estimated marginal means show higher reduction (M = 4.79) in CDT group than CT group (M = 3.81). The pairwise comparison shows that the mean difference was high between baseline and 4th week measurement of VAS (3.03) than between 4th week and 6th week (1.68). So it was inferred that CDT was effective in reducing the pain in comparison to CT and the time series repeated measure show that therapy was more effective in the first four weeks of intervention.

**Table 3:** Test of within subjects effects for all outcome measures

	F Value					
Source	VAS	EORTC QLQ C30 Global scale	EORTC QLQ C30 Functional scale	EORTC QLQ C30 Symptoms scale	EORTC QLQ BR23 Functional scale	EORTC QLQ BR23 Symptoms scale
Time	991.96*	120.36*	511.72*	1114.05*	339.71*	13.13*
Time*Group	29.34*	55.91*	120.09*	294.80*	92.87*	2.60*

*VAS* visual analogue scale, *Statistically significant (*p* < 0.05)

**Table 4:** Test of between subjects effects for all measures

	Groups	Mean(range) (95 % CI)	Mean differences (95 % CI)	F
VAS	CDT	3.81 (3.53–4.10)	0.98 (0.58–1.38)	23.82*
	CT	4.79 (4.51–5.07)		
EORTC QLQ C30 Global scale	CDT	43.54 (41.88–45.21)	2.97 (0.61–5.32)	6.36*
	CT	40.58 (38.91–42.24)		
EORTC QLQ C30 Functional scale	CDT	82.82 (82.26–83.38)	1.94 (1.15–2.74)	24.06*
	CT	80.89 (80.32–81.44)		
EORTC QLQ C30 Symptoms scale	CDT	44.02 (42.81–45.24)	2.10 (0.38–3.82)	6.01*
	CT	41.92 (40.71–43.14)		
EORTC QLQ BR23 Functional scale	CDT	35.57 (34.76–36.37)	2.38 (1.24–3.52)	17.47*
	CT	33.19 (32.38–33.99)		
EORTC QLQ BR23 Symptoms scale	CDT	61.18 (60.14–62.22)	1.52 (0.05–2.10)	4.28*
	CT	59.66 (58.61–60.70)		

*CT* conventional therapy, *CDT* complete decongestive therapy, *VAS* visual analogue scale, *Statistically significant (*p* < 0.05)

### EORTC QLQ C30

**Fig. 2:**
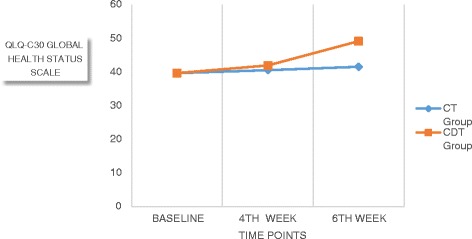
Comparison of QLQ C-30 (Global health status scale) values within and between the groups

**Fig. 3:**
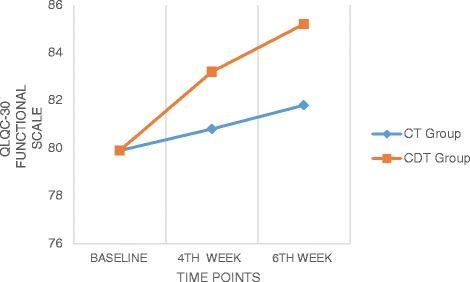
Comparison of QLQ C-30 (Functional scale) values within and between the groups

**Fig. 4:**
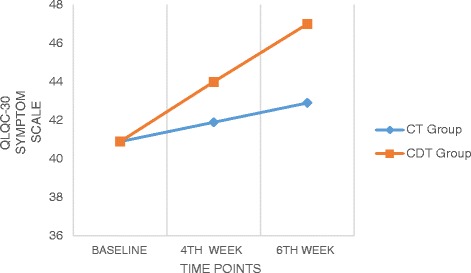
Comparison of QLQ C-30 (Symptom scale) values within and between the groups

The values of QLQ-C30 global health status scale, functional and symptom scales at baseline, 4th and 6th weeks were represented in Figs. 2, 3 and 4 respectively.

Significant differences were observed across the time factor for global, functional and symptoms scales of the EORTC QLQ C30, and F values were 120.36, 511.72 and 1114.05 (*p* < 0.05) respectively (Table 3). Between groups factor was significantly different in all these three sub scales with F value of 6.36 for global scale, 24.06 for functional scale and 6.01 for symptoms scale at *p* < 0.01 (Table 4). There was also a significant interaction between time and group, *F* = 55.91, 120.09, and 294.80 (*p* < 0.05) for all the sub scales. The estimated marginal means show that CDT group had significantly higher improvement than the CT group and the pairwise comparison shows that the mean difference was high between baseline and 4th week measurement for all the scales (4.12, 2.13 and 2.07) than between 4th week and 6th week (1.63, 1.48 and 1.98).

### EORTC QLQ BR 23

**Fig. 5:**
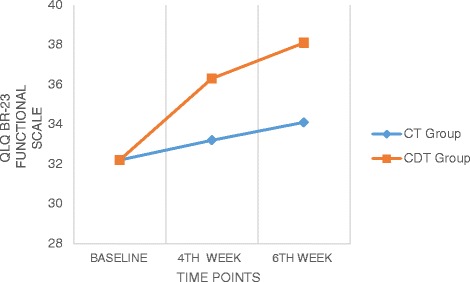
Comparison of QLQ BR-23 (Functional scale) values within and between the groups

**Fig. 6:**
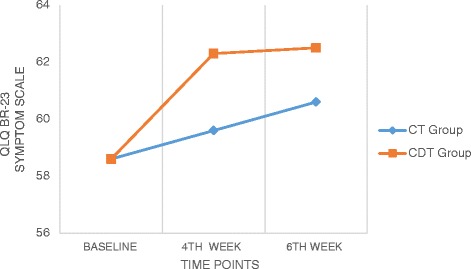
Comparison of QLQ BR-23 (Symptom scale) values within and between the groups

Analysis of EORTS QLQ BR 23 measures showed significant differences across the time factor for both the functional and symptoms scales with an F values of 339.71 and 13.13 (*p* < 0.05) respectively (Table 3). Between subjects factor were also significantly different in all there three scales with F value of 17.47 for functional scale and 4.28 for symptoms scale (*p* < 0.01) (Table 4). There was also a significant interaction between time and group, *F* (2116) = 92.87 and 2.60 for functional and symptoms scales of the EORTS QLQ BR 23 questionnaire. The estimated marginal means show that CDT group had significantly higher improvement than the CT group in all the scales (Figs. 5 and 6). The pairwise comparison shows that the mean difference was high between baseline and 4th week measurement for both the sub scales (2.58 and 2.33) than between 4th week and 6th week (1.37 and 0.58).

## Discussion

The findings of this study indicate that subjects in both groups showed improvement in pain (VAS) and QOL as measured by QLQ-C 30 213 and QLQ-BR 23. However, there was more improvement with CDT and home program. Comparing the effectiveness of the above treatment strategies in subjects with post mastectomy lymphedema (PML), CDT group showed improved QOL and significant reduction in pain. Greatest reduction in pain leading to improved quality of life in first 4 weeks of treatment, with the effect continuing at a slower rate in next two weeks. In this study we found CDT and remedial exercises along with a one hour home program helped to improve QOL. Kim SJ et.al [43] studied the effect of complex decongestive therapy on unilateral lymphedema and quality of life in breast cancer patients. The results showed that QOL significantly improved by reduction of upper limb lymphedema during the maintenance phase, which was correlated with the reduction in limb volume and significant difference in physical functioning and quality of life as measured by Korean version of SF-36 health survey [41, 42, 44].

Karadibak D et.al [42] conducted a prospective trial of intensive decongestive physiotherapy for upper extremity lymphedema and showed decrease in edema, fear of activity, and improved quality of life. To date, several studies have been published investigating the effects of CDT and MLD in preventing and treating post mastectomy lymphedema [45–51]. Badger et al. [52] in 2004 conducted a randomized clinical trial that tested physical therapies with a follow-up period of at least 6 months. They concluded wearing a compression sleeve is beneficial and bandage-plus hosiery resulted in a greater reduction in excess limb volume than hosiery alone.

Previous studies on the effectiveness of treatment for breast cancer related lymphedema found that CDT was effective but failed to demonstrate the unified approach to treatment. Most of the study populations are specific and have methodological limitations such as lesser sample sizes, lack of control groups etc. Hence, researchers concluded that long term follow up is needed to demonstrate the relative contribution of individual components such as compression bandaging, exercises, MLD etc. [53]. In contrast, our study proved the relative contribution of remedial exercises and a one hour home program in reducing lymphedema and enhancing QOL. Meneses and McNees [54] in 2007 cited 86 articles in their review and showed reduced QOL with lymphedema and concluded that CDT appears to be helpful in treating lymphedema. Our findings that greater improvement in the pain, symptoms and function in first 4 weeks than between 4th and 6th weeks was consistent with another study showing maximum reductions achieved in first few days of treatment, with improvement continuing at a slower rate in the next weeks [55]. Martin et al. [56] provided information on the effectiveness of MLD and its impact on the quality of life and physical limitations among PML patients. They used EORTC QLQ-C30 version 2.0 for cancer in general and EORTC QLQ-BR23 specific for breast cancer to assess the efficacy of the treatment, measuring the improvement of the lymphedema. However, these studies have been inconclusive, and accurate information on health-related quality of life (HRQOL) outcomes among post mastectomy lymphedema (PML) patients is needed. In the present study, EORTC QLQ-C30 version 3.0 for cancer in general and EORTC QLQ – BR 23 specific for breast cancer was used which yielded better results than the previous study because of inclusion of home program with emphasis on remedial exercises. It is important since lymphedema is known to have a significant impact on the physical, psychological, and social well-being of the patients. In the present study the participants’ cooperation and adherence to home program were high. This may be due to the attention given to them by the researchers. Therefore, the present study emphasized the role of remedial exercises and a home program in addition to CDT is more beneficial in health, function and symptom status of QOL in post mastectomy lymphedema patients.

## Conclusion

Clinically and statistically relevant improvement in QOL was observed in the CDT group who received remedial exercises and home program in addition to compression bandage and manual lymphatic drainage. Therefore, remedial exercises and home program should be incorporated in the treatment protocol of breast cancer related lymphedema patients.

### Limitations of the study

The first limitation of the study was the small sample size. Secondly, there was no follow up to observe the long term effects of this technique. Assigning participants to treatment group based on the day of the week they are seen in clinic can introduce biases and lack of random assignment would be a potential limitation of this study. Future works are required to evaluate the outcome of a unified plan of treatment in specific population for the greater generalizability of the outcomes.

## Abbreviations

CDT: complete decongestive therapy
CT: conventional treatment
MLD: manual lymphatic drainage
PML: post-mastectomy lymphedema
QOL: quality of life
